# Evidence That the Adenovirus Single-Stranded DNA Binding Protein Mediates the Assembly of Biomolecular Condensates to Form Viral Replication Compartments

**DOI:** 10.3390/v13091778

**Published:** 2021-09-06

**Authors:** Paloma Hidalgo, Arturo Pimentel, Diana Mojica-Santamaría, Konstantin von Stromberg, Helga Hofmann-Sieber, Christian Lona-Arrona, Thomas Dobner, Ramón A. González

**Affiliations:** 1 Centro de Investigación en Dinámica Celular, Instituto de Investigación en Ciencias Básicas y Aplicadas, Universidad Autónoma del Estado de Morelos, Cuernavaca 62209, Mexico; dianamojicaa@gmail.com (D.M.-S.); chris.arrlo@hotmail.com (C.L.-A.); 2 Leibniz Institute for Experimental Virology (HPI), 20251 Hamburg, Germany; konstantin.stromberg@leibniz-hpi.de (K.v.S.); Helga.hofmann-sieber@gmx.de (H.H.-S.); thomas.dobner@leibniz-hpi.de (T.D.); 3 Laboratorio Nacional de Microscopía Avanzada (LNMA), Instituto de Biotecnología, Universidad Nacional Autónoma de México (UNAM), Cuernavaca 62210, Mexico; arturo.pimentel@ibt.unam.mx; 4 Instituto de Biotecnología, Universidad Nacional Autónoma de México, Cuernavaca 62210, Mexico

**Keywords:** human adenovirus type 5 (HAdV-5), ssDNA-binding protein (DBP), liquid-liquid phase separation (LLPS), biomolecular condensates (BMCs), virus-induced cellular compartmentalization, Replication Compartments (RCs)

## Abstract

A common viral replication strategy is characterized by the assembly of intracellular compartments that concentrate factors needed for viral replication and simultaneously conceal the viral genome from host-defense mechanisms. Recently, various membrane-less virus-induced compartments and cellular organelles have been shown to represent biomolecular condensates (BMCs) that assemble through liquid-liquid phase separation (LLPS). In the present work, we analyze biophysical properties of intranuclear replication compartments (RCs) induced during human adenovirus (HAdV) infection. The viral ssDNA-binding protein (DBP) is a major component of RCs that contains intrinsically disordered and low complexity proline-rich regions, features shared with proteins that drive phase transitions. Using fluorescence recovery after photobleaching (FRAP) and time-lapse studies in living HAdV-infected cells, we show that DBP-positive RCs display properties of liquid BMCs, which can fuse and divide, and eventually form an intranuclear mesh with less fluid-like features. Moreover, the transient expression of DBP recapitulates the assembly and liquid-like properties of RCs in HAdV-infected cells. These results are of relevance as they indicate that DBP may be a scaffold protein for the assembly of HAdV-RCs and should contribute to future studies on the role of BMCs in virus-host cell interactions.

## 1. Introduction

Viruses account for up to 80% of all acute respiratory infections [1]. Human adenoviruses (HAdVs) from species B, C and E are the causative agents of 5–10% of acute respiratory infections in children [2,3], and can cause pneumonia, which can be fatal not only in immunocompromised patients—where the fatality rate can be 50% or higher—but also in healthy children and adults [4]. Although HAdVs are clinically relevant viruses, there are no specific treatments, or vaccines available to the general public. Besides their relevance as human pathogens, adenoviruses are one of the most commonly used viral vectors in the design and development of vaccines, as well as gene and combined anti-cancer therapies [5,6]. Moreover, adenoviruses have been used as a model system to study eukaryotic cellular processes, such as transcription, post-transcriptional processing, nuclear organization, cell cycle control and cell death [7,8].

During infection of permissive cells, HAdVs induce alterations of the host-cell gene expression program and the cell cycle [9,10,11,12,13]. Viral replication is accompanied by an extensive intranuclear rearrangement of cellular factors that are major constituents of nuclear domains (ND). Examples of such ND include the promyelocytic leukemia protein (PML)-Nuclear Bodies (PML-NB), the nucleolus, Cajal Bodies (CB) and Interchromatin Granules (IG) [14,15,16,17,18,19,20,21,22], which harbor proteins that participate in a variety of cellular activities, from the DNA damage response (DDR) to regulation of gene transcription and posttranscriptional processing of RNA. In HAdV-infected cell nuclei multiple components of ND are relocalized to sites where the viral DNA, viral RNA and viral proteins accumulate, presumably resulting in the assembly of viral Replication Compartments (RCs) where the viral genome is replicated and viral genes are expressed. Interestingly, cellular proteins involved in pathways that would otherwise restrict viral replication are co-opted and subverted in RCs (reviewed in [23]). Virus-induced compartmentalization of the cell is not an exclusive replication strategy of HAdVs. Assembly of compartments termed “viral organelles” or “factories”, “inclusion bodies” or “viroplasms” have been described for other viruses as sites for viral genome replication that concomitantly serve to conceal the viral genome from detection by cellular antiviral mechanisms [24,25,26,27,28]. Additional events of the viral replication cycle, such as viral gene transcription and RNA posttranscriptional processing, as well as the assembly of virus progeny, may also be coordinated in virus-induced compartments [23,28,29,30,31,32]. Therefore, although different virus families have very different genome structure and replication strategies, it is of relevance that they all may induce compartmentalization of the infected cell to promote virus replication.

Compartmentalization is essential for the organization of cellular functions and to maintain cellular homeostasis. Formation of cellular compartments is achieved either through delimitation of subcellular spaces by membranes, as is the case of the membrane-bound cellular organelles, or through formation of membrane-less biomolecular condensates (BMCs) which are commonly formed by liquid-liquid phase separation (LLPS) [33]. During LLPS, the rise in concentration and in weak multivalent interactions between groups of macromolecules in a well-mixed solution separates from the surrounding cellular milieu, resulting in the formation of defined liquid condensates, such as the cytoplasmic RNA granules and various nuclear bodies [34,35]. These membrane-less compartments have been referred to as BMCs to unify their common features of concentrating biomolecules and assembling by LLPS, regardless of their intracellular localization, composition or activities [36]. BMCs are highly dynamic, with heterogenous stoichiometry, constantly interchanging components with the surrounding space, and are mainly composed of proteins that establish transient multivalent interactions with nucleic acids. Such multivalent interactions are promoted by low-complexity regions (LCRs), intrinsically disordered regions (IDRs) and nucleic-acid binding domains, and are often regulated by post-translational modifications (PTMs) [37,38]. The defining properties of BMCs that form by LLPS are that liquid condensates can fuse and divide, rapidly reorganize internally, respond to stimuli and age or mature into gel-like or solid states [39,40]. LLPS has been reported as a mechanism for the assembly of virus-induced intracellular compartments for viruses of the order *Mononegavirales*, for the dsRNA rotaviruses, influenza viruses, coronaviruses, retroviruses and herpesviruses [29,41,42,43,44,45]. Detailed insights into mechanisms of virus-induced assembly of cellular compartments is paramount to understand essential aspects of their biology, to learn how viruses hijack infected cells and to study fundamental mechanisms that may be common in virus-host cell interactions, which should in turn help to develop antiviral strategies and improve the use of viruses as vectors for vaccines or gene and anticancer therapies [46,47].

HAdV possess a linear dsDNA genome of approximately 36 kbp that encodes the proteins required for viral DNA replication, namely the preterminal protein primer (pTP), the DNA polymerase and the ssDNA binding protein (DBP). The latter participates in strand-displacement during viral DNA synthesis through cooperative binding of ssDNA [48]. The DBP is a multifunctional protein of 529 amino acid (aa) residues that has the ability to bind both DNA and RNA [49,50,51,52], and in addition to its role in viral DNA synthesis, it promotes gene expression and is essential for viral growth [53,54]. The N-terminal domain of DBP is subjected to phosphorylation and SUMOylation, PTMs that modulate the protein’s intracellular localization and activities [55,56]. DBP is one of the main components of RCs and therefore has been used as a marker for the study of these viral structures [23].

Like membrane-less organelles and ND, HAdV-RCs are not membrane-enclosed nuclear assemblies, largely formed by nucleic acids and proteins that dynamically interchange components with the surrounding milieu [23,30,57]. These features led us to hypothesize that the driving force for RC-formation could be LLPS. In the present work, we show that the HAdV-RCs can be described as viral BMCs with liquid-like properties that can be conferred by DBP. This early multivalent viral protein is capable of driving protein condensation both in infected and in transfected cells, indicating that DBP may be a scaffold protein of RCs. We show that early and intermediate DBP-positive RCs have diffusion coefficients similar to other cellular and viral phase-separated organelles, with the ability to fuse to each other and undergo maturation to a less liquid-like state at late times post-infection, when they become increasingly subcompartmentalized. Taken together, the evidence indicates that the DBP has liquid-like properties that mediate the assembly of viral BMCs and suggest that viral RCs may be assembled by LLPS.

## 2. Materials and Methods

### 2.1. Cells

Primary human foreskin fibroblasts (HFF; kindly provided by Jesús Santa-Olalla (School of Medicine—UAEM)) were maintained in monolayer cultures in Dulbecco’s modified Eagle’s medium (DMEM, Gibco; Carlsbad, CA, USA) supplemented with 10% (*v/v*) fetal bovine serum (FBS, Gibco), 100 U/mL penicillin and 100 µg/mL streptomycin (Gibco) for no more than 10 passages. H1299 cells (ATCC no. CRL-5803: ATCC Global Bioresource Center, Manassas, Virginia, VA, USA) were maintained in monolayer cultures in DMEM supplemented with 10% (*v/v*) FBS, 100 U/mL penicillin and 100 µg/mL streptomycin. All cells were kept at 37 °C in 5% CO_2_. HFF were infected at 30 FFU/cell, as described previously [58].

### 2.2. Viruses

The viruses used in this work were the phenotypically wild-type (WT) HAdV-C5 (H5pg4100, HAdV5 WT) [59,60] and a virus with the H5pg4100-backbone that encodes an N-terminally mCherry-tagged DBP (H5hh4300, hereafter called mCherry-DBPv), generated as described previously [61] using homologous recombination and ccdB counterselection [62].

### 2.3. Antibodies

The primary antibodies used included mouse monoclonal Ab (mAb) B6-8 [63] against DBP and mouse mAb against β–actin (AC-15, Sigma Aldrich, MO, USA). Secondary antibodies included HRP-anti-mouse IgG (Jackson ImmunoResearch, Cambridge, UK) and Alexa Fluor 488 or 555 anti-mouse IgG (Invitrogen, Waltham, MA, USA).

### 2.4. Plasmids

The vector mCherry-C1 was constructed by removing the CFP cassette from the pECFP-C1 vector using NheI and XhoI and replaced with the mCherry cassette. The E2A gene was cloned in frame at the 3′ end of the mCherry coding sequence between the XhoI and EcoRI sites to construct a plasmid that encodes an N-terminally mCherry-tagged DBP (mCherry-DBPp). The gene construct includes a 12 nucleotide linker sequence which encodes the aminoacid sequence RSRA between the mCherry open reading frame and that of the E2A gene. The pCMX3b-Flag E2A used in transient transfections experiments encodes an N-terminally FLAG-tagged DBP [55].

### 2.5. Prediction Software

The DBP sequence (UniProt ID: P03265) was analyzed using the following software: For protein disorder prediction, PONDR pool [PONDR VLXT, PONDR XL1_XT, PONDR VL3-BA, PONDR VSL2] (www.pondr.com, accessed on 20 December 2020) and IUPRED 2 (https://iupred2a.elte.hu, accessed on 20 December 2020); for LCR prediction, Motif Scan (MyHits, Swiss Institute of BioinformaticsIB, Switzerland); to determine the predicted propensity of DBP to induce phase separation, PSPredictor [64].

### 2.6. Click-Chemistry to Label Newly Synthesized Viral DNA

The Click-iT 5-ethynyl-2′-deoxyuridine (EdU) Cell Proliferation Kit for Imaging, with Alexa Fluor 488 dye (ThermoScientific, Waltham, MA, USA), was used to label the viral DNA, according to the manufacturer’s instructions. Briefly, HFF cells were grown on coverslips and were infected at a confluence of 80% with the mCherry-DBPv virus. To favor EdU incorporation into viral DNA over host DNA, EdU was added to the culture medium at 20 h post-infection (hpi), when an exponential increase in viral DNA synthesis is observed and cellular DNA replication is mostly abrogated in HAdV-infected HFF cells [58]. Cells were fixed at 28 hpi and subjected to copper (I)-catalyzed click reaction to conjugate EdU with the azide-Alexa Fluor 488 dye. The coverslips with the cells were mounted on glass slides in PBS/10% glycerol, sealed with clear nail polish and samples were analyzed using a Zeiss Axiovert 200 M inverted microscope, equipped with a 63×/1.4 numerical aperture oil-immersion objective lens, with an Axiocam MRM and Axiovision 3.1 software (Carl Zeiss, Inc., Oberkochen, Germany).

### 2.7. Western Blot Analyses

To analyze the steady-state concentrations of DBP, HAdV-infected cell pellets were incubated in radioimmunoprecipitation assay (RIPA) lysis buffer on ice for 30 min, vortexed every 10 min. Total cell lysates were sonicated (Branson Ultrasonics, Brookfield, CT, USA) using 40 pulses (output 0.8), centrifuged (13,000 rpm, 3 min, 4 °C) and the supernatant (SN) was recovered. Protein concentration was measured spectrophotometrically using Bradford reagent (Bio-Rad, Hercules, CA, USA). For immunoblotting, 50 µg of total protein lysates were analyzed by SDS-PAGE, transferred onto nitrocellulose membranes (GE Healthcare, Chicago, IL, USA) and blocked over-night with 5% non-fat milk. The next day, the membranes were washed and incubated for at least 2 h at 4 °C with primary antibodies (B6-8, 1:10; anti-actin 1:1000). After successive washes with PBS/0.1% Tween20 (PBS-T), the membranes were incubated with secondary antibodies coupled to HRP for at least 45 min at 4 °C and thoroughly washed with PBS-T. Membranes were developed by enhanced chemiluminescence as recommended by the manufacturer (Pierce, Thermo Scientific, Waltham, MA, USA) and bands were visualized on X-ray films (Fujifilm Corporation, Minato, Tokyo, Japan).

### 2.8. Transfection of mCherry-E2A in H1299 Cells

H1299 cells were transfected with the indicated amounts of the plasmids, using polyethylenimine (PEI) at a 10:1 ratio (PEI (µg):total DNA (µg)). The transfection medium was replaced with DMEM, 10% FBS, 100 U/mL penicillin and 100 µg/mL streptomycin after 6 h and cells were analyzed 24 h post-transfection (hpt).

### 2.9. Time-Course Microscopy of Fixed Cells

An Olympus IX-81 inverted fluorescence microscope (Olympus, Shinjuku, Tokyo, Japan) was used. The samples were continuously illuminated using 405 nm or 488 nm diode-pumped solid-state lasers. Beam selection and modulation of laser intensities were controlled via Xcellence software v.1.2, Olympus soft imaging solution GMBH. A full multiband laser cube set was used to discriminate the selected light sources (LF 405/488/561/635 A-OMF, Bright Line; Semrock, NY, USA). Fluorescence was collected using an Olympus UApo N 100×/1.49 numerical aperture oil-immersion objective lens with an extra 1.6× intermediate magnification lens and an EMCCD camera (iXon 897, Model No: DU-897E-CS0-#BV; Andor, Belfast, UK). A collection of 40 Z-stacks spaced every 0.16 µm were obtained per each time-point. The micrographs were processed by deconvolution using the classical maximum likelihood estimation method (CMLE) with a total of 30 iterations using the Huygens 2018 software at 8-bits, with a pixel size of 100 nm and a resolution of 600 dpi.

### 2.10. Confocal Microscopy of Transiently Transfected Fixed Cells

H1299 cells were seeded on coverslips, transfected at a confluence of 70% with the specified amount of plasmid DNA and imaged 24 hpt using an inverted confocal scanning laser microscope (Nikon A1R HD25 equipped with a Nikon 60× oil-immersion NA 1.40 objective; Nikon, Minato, Tokyo, Japan). Images were acquired as 22 stacks spaced every 0.3 µm. Images were processed using Fiji [65] and are presented as maximum intensity projections.

### 2.11. Fluorescence Recovery after Photobleaching (FRAP) Image Acquisition

For FRAP analysis of infected cells, HFF cells were plated on glass-bottom culture dishes (35 mm Fluorodish, World Precision Instruments, Sarasota, FL, USA), infected at a confluence of 80% with the mCherry-DBPv virus and analyzed at 16, 24 and 36 hpi. Data acquisition was performed on an FV1000 Olympus inverted confocal microscope IX81 with a UPLSAPO 60X S2 NA 1.3 oil immersion objective, a 543 nm laser line and an incubator with controlled temperature (37 °C) and CO_2_ (5%). Bleaching was performed with a 405 nm laser line laser at 100% power. At least 16 cells per condition were analyzed. Data from the FRAP analyses and parameters calculated can be found in the Supplementary Data, Tables S1–S3 and Figures S1–S3.

For FRAP analysis of transfected cells, H1299 cells were plated on glass-bottom culture dishes (μ-Dish 35 mm, Ibidi. Gräfelfing, Germany), transfected at a confluence of 70% with the specified amounts of mCherry-DBPp plasmid and imaged 24 hpt using an inverted confocal scanning laser microscope (Nikon A1R HD25 equipped with a Nikon 60× oil-immersion NA 1.40 objective) and an incubator with controlled temperature (37 °C) and CO_2_ (5%). Bleaching was performed with a 561 nm laser line laser at 25% power. The same laser line was used for bleaching and for imaging). At least seven cells per condition were analyzed. Data from the FRAP analyses and parameters calculated can be found in Supplementary Data, Table S4 and Figure S4.

### 2.12. FRAP Image Processing and Analysis

The images were corrected for x/y drift using the Fiji plugin Template Matching [65]. The recovery curve was calculated taking into consideration three circular regions of interest (ROIs): the bleached region (ROI1), the reference region (ROI2, a fluorescent non-bleached region) and the background region (ROI3). For each single frame of the time series collection the mean fluorescence intensity for all ROIs were measured, including before, during and after bleaching. The average of the ROI3 signal was subtracted from ROI1 and ROI2 to generate background corrected versions. The mean intensity of ROI1 was normalized and corrected (to consider photobleach by observation and defocus) using the intensity time series obtained from ROI2. The normalized recovery curve of the fluorescence intensity, named *I*(*t*), was obtained. The recovery fraction (RF) and the halftime of recovery (*τ*_1/2_) were calculated according to the following procedure.

The time series *I*(*t*) was described by an exponential function:

(1) $$I\left(t\right)=\alpha e^{\beta t}+\theta$$

where *α*, *β* and *θ* are coefficients fitted by a non-linear square method and *t* is the time.

For a normalized recovery curve, the *τ*_½_ is given by

(2) $${I(\tau }_{1/2})=\frac{\ln\;(1/2)}{\beta }$$

where *β*, is the fitted coefficient of Equation (1) [66,67,68].

According to [69], under a unidimensional diffusion model without borders, the characteristic diffusion time$\;\tau_{D\;}$is defined by the expression:

(3) $${\;\tau }_{D\;}=\frac{r^{2}}{4\;D}$$

where *D* is the lateral diffusion coefficient of the fluorescently labelled molecules and *r* is the radius of the bleached spot (ROI1). This expression can be related to τ_½_ according to the following equation:

(4) $$\tau_{1/2}{=\gamma \;\tau }_{D\;}=\frac{{\gamma \;r}^{2}}{4\;D}\;$$

where γ is a parameter which depends on the bleaching percentage. This parameter takes into consideration the effective bleached area and its dependence on the bleached fluorescence percentage. The value of γ was obtained from [69]. Image processing and calculation of parameters were performed in Fiji and R [70], respectively. Calculated parameters as well as time-series plots can be found in Supplementary data.

### 2.13. Spinning-Disc Confocal Live-Cell Microscopy and 3D Time-Lapse Assays

HFF cells infected with the mCherry-DBPv virus were visualized from 20 to 24 hpi. Data acquisition was performed on a CSU-W1 Yokogawa SDC on an inverted Zeiss microscope Observer Z1 equipped with Plan Neo 20×/0.8 NA, Plan Apo 63×/1.4 N.A. The fluorophore was excited with the 561 nm (20 mW) line diode laser combined with a BrightLine Emission filter of 617/73 nm. Images were acquired with an Andor iXon 5078 controlled with Slide Book 6.17 software. Multiple stage positions were collected using a WK-XYBH-APZ30-AV00FT ASI stage controller and optical sections were collected using a Z-stage ASI Piezo MS-2000 Controller. Environmental conditions were controlled by an Okolab H301-K-Frame incubator placed into a Cell Okolab Observer SD enclosure. The set was adjusted to 37 °C and 5% CO_2_. A single stack was composed of 37 frames. Each frame (optical slice) had a dimension of 130 µm × 130 µm in XY and 0.27 µm in Z. The Z step between frames was the same than the optical width (0.27 µm). The whole stack acquisition took 7.4 s and the time between stacks (the first frame of one stack and the first frame of the next stack) was 5 min. In all, 13 stacks (timepoints) were acquired and cell focus was maintained throughout the whole acquisition.

### 2.14. Stacks Processing and Quantification

Acquired stacks were processed using the software Fiji according to the following procedure. A stack is composed of a set of 37 planes separated by 0.27 µm. Each single plane is composed of 512 × 512 voxels (130 × 130 × 0.27 µm^3^) with physical sizes of 0.2539 × 0.2539 × 0.2699 µm^3^ before deconvolution and 0.2539 × 0.2539 × 0.135 µm^3^ after the deconvolution process. The deconvolved stack is cropped to reduce the analysis region to the contained cell. Then, using the Image → Scale tool of Fiji, a bicubic interpolation increases, by five times, the number of voxels into the image. This procedure does not increase the real physical resolution of the image, but the implicit average of the interpolation operation improves the SNR with the consequent positive effects into the 3D measurements and the ensuing 3D rendering. The gray levels of interpolated data for the whole stack were equalized using the Image → Adjust → Brightness and Contrast tool, using the auto option. The obtained outputs were used as input into the Analyze → 3D Object Counter Plugin in Fiji [71]. The reported parameters include volume and surface quantifications. For each single object, given its volume and surface, the sphericity index was calculated as the ratio of the nominal surface area, S_n_ (surface area of a sphere having the same volume as the object), to the actual surface area of the object, *S*, according to the following equation:

(5) $$\psi =\frac{\sqrt[{3}]{36\;\pi \;V^{2}}\;}{S}$$

where *V* is the measured volume of the object. The index ranges from 1 (perfect sphere) to 0 (elongated shape) [47].

### 2.15. Stacks 3D + t Rendering

The processed stacks referred in the previous section were rendered using Fiji according to the following procedure. Once cropped and deconvolved, the stacks were slightly smoothed by a Gaussian blur filter tuned with a radius of 0.8 µm. Then, using the Image → Scale tool of Fiji, a bicubic interpolation tripled the number of voxels in the image. Finally, after a brightness and contrast auto adjustment, using the Plugins → 3D viewer option, the 3D + t rendering was performed. By means of the 3D viewer options and the lookup tables (LUT) tools, the movies of the supplementary material were generated.

## 3. Results

### 3.1. DBP-Positive RCs Resemble BMCs

**Figure 1:**
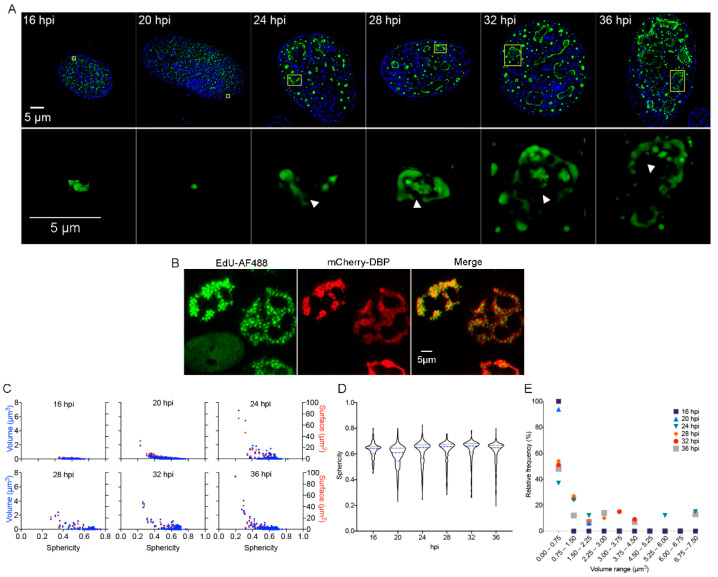
DBP-positive RCs resemble BMCs as they increase in size and shape complexity and appear increasingly subcompartmentalized with progression of the replication cycle. (**A**) HFF cells were infected at a MOI of 30 FFU/cell with HAdV5 WT and fixed at the times indicated. Cells were imaged by acquisition of Z-stacks as described in Materials and Methods to study the intranuclear distribution of DBP by indirect immunofluorescence using the B6-8 primary antibody and anti-mouse Alexa Fluor 488-IgG secondary antibody. Mock-infected cells were included and are shown in the Supplementary Materials. (A-upper panels) Images from whole cells are presented as deconvoluted maximum intensity projections of DBP (green) and DAPI (blue). (A-lower panels) Insets show a 2D perspective of 3D reconstructions generated from the corresponding Z-Stack (indicated in yellow boxes in the upper panels) that illustrate the increase in complexity of DBP-assemblies with progression of the replication cycle. The arrowheads point to subcompartmentalized, hollow DBP-assemblies. The images were extracted from Supplementary Movies S1–S6. The color palette corresponds to the pixel gray level intensity under the Lookup Table Green (Fiji). (**B**) HFF cells were infected with the mCherry-DBPv virus, incubated with EdU at 20 hpi and fixed 8 h later, as described in Materials and Methods. A copper (I)-catalyzed click reaction was performed to conjugate EdU with Alexa Fluor 488 dye (AF488) to stain the sites of accumulation of newly replicated viral DNA. Note that the non-infected cell (absence of mCherry-DBP signal in the lower left corner) has a diffuse staining for AF488 compared to the foci distribution in infected cells (mCherry-DBP positive). (**C**) Dot-plots comparing the volume (blue dots) and surface (red dots) as a function of the sphericity index of DBP-assemblies at different time-points. (**D**) Violin plots of the sphericity index as a function of different time-points. The sphericity index ranges from 1 (perfect sphere) to 0 (non-spherical). (**E**) Dot-plot representing the relative frequency of DBP-assemblies grouped in different volume ranges for each time-point.

In HAdV-infected cells DBP forms defined nuclear foci termed ‘early replicative sites’ where viral ssDNA and dsDNA, as well as viral early unspliced and spliced mRNAs, are localized [7,17,23,72,73,74]. This process is initiated as soon as this viral early protein is imported into the nucleus during the early phase of viral replication, when the protein is distributed in small intranuclear foci (Figure 1A, 16–20 hpi).

As the viral replication cycle progresses and viral genome replication begins, DBP organizes in slightly larger spheroid foci and ring-like structures (Figure 1A, from 20 to 24 hpi) that ultimately form more complex morphologies as the ring-like structures coalesce at late times of infection (Figure 1A, 28–36 hpi) [75]. At the onset of viral genome replication (approximately 20 hpi in HAdV-infected HFF cells), RCs display two prominent shapes: the spheroid morphology that is similar to the foci observed from the earlier time-points (16 hpi) and throughout the replication cycle, and the more complex ring-like or amorphous structures that appear after 20 hpi (Figure 1A upper and lower panels). The latter seem to originate from the fusion of two or more ring-like structures, when they appear to form subcompartmentalized, seemingly hollow DBP-assemblies (Indicated by arrowheads in Figure 1A, lower panels and supplementary movies 3–6). By 36 hpi, DBP-spheroid RCs can still be detected; however, multiple DBP-assemblies appear to coalesce, forming a reticulated meshwork that occupies most of the nuclear volume (Figure 1A, 36 hpi.). Using click-chemistry and the incorporation of the EdU nucleotide analog, the sites of accumulation of newly replicated viral DNA were visualized within DBP assemblies, suggesting that the subcompartments are sites of viral DNA synthesis (Figure 1B). These sites have also been reported as positive for pTP-staining [76], lending support to our observation.

In order to study in more detail the distribution of DBP in the infected cell-nucleus, quantitative analyses were performed using the data obtained directly from the Z-stacks (without rendering) to calculate the volume, surface and sphericity index of the DBP-structures. The volume (blue dots) and surface (red dots) were plotted as a function of the sphericity index (Figure 1C). The distribution of the sphericity as a function of the different time-points is represented by violin plots (Figure 1D). As expected, at 16 hpi, all DBP-assemblies were small structures with a spheroid shape, and at later times post-infection, DBP-positive structures increased in size and deviated from a spherical shape (Figure 1D, 20–36 hpi and 1E).

At 16 and 20 hpi, almost 100% of the DBP-assemblies had volumes between 0.00037 and 0.75 µm^3^. The percentage of structures within this size range decreased to 40–60% starting from 24 hpi. At later time-points, although most of the structures displayed a small size, approximately 20% displayed a volume of up to 7.5 µm^3^ (Figure 1E). These results indicate that formation of DBP-assemblies resembles other reported cellular and viral BMCs and suggest that RCs coalesce through fusion of smaller condensates, forming assemblies that are larger with a subcompartmentalized appearance.

### 3.2. DBP Assembles BMCs with Liquid-Like Properties at Early Times Post-Infection That Mature with Progression of the Viral Replication Cycle to Form Less Fluid-Like Condensates

Since various viral replication organelles have been recently reported to display liquid-like properties and contain components that may induce phase transitions, and since the distribution of DBP throughout the infection resembles that of liquid-like BMCs (Figure 1), we hypothesized that DBP may have properties that allow condensation and assembly of viral replication compartments with liquid-like characteristics.

**Figure 2:**
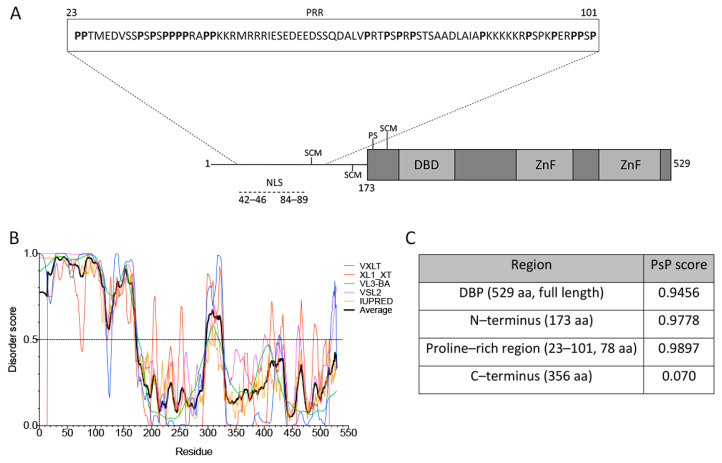
DBP has sequence features of proteins that drive phase transitions. (**A**) Schematic representation of the HAdV-5 DBP. The N-terminal line represents the predicted intrinsically disordered region (IDR) from aa residues 1–173. The protein crystal has been resolved for aa residues 174–529 (gray boxes), showing that the central region forms a globular core which is composed of α-helices and ß-sheets, and a flexible hinge at the C-terminus. The DNA binding domain (DBD), zinc-fingers (ZnF), the known SUMO conjugation motifs (SCM) and phosphorylation sites (PS) as well as the nuclear localization signal (NLS) are indicated. The inset shows the proline-rich region (PRR) from aa residues 23–101, as predicted by Motif Scan. (**B**) The VXLT, XL1_XT, VL3-BA, VSL2, and IUPRED predictors were used for protein disorder analysis. An IDR was consistently predicted at the N–terminus (disorder score above 0.5). The black line represents the average score of all six algorithms for each amino acid residue. (**C**) PSPredictor (PSP) scores for different domains and motifs of DBP. PSPredictor scores from 0–1, a value above 0.5 indicates the peptide/protein is predicted to induce phase separation.

The structure of DBP from aa residues 174–529 contains a globular core, composed of α-helices and β-sheets, but also a flexible hinge that facilitates different degrees of rotational freedom to bind nucleic-acids with no sequence specificity (represented in Figure 2A, gray boxes) [48,77].

The lack of a crystal structure for the N-terminus (aa residues 1–173) coincides with the sequence analysis using the VXLT, XL1_XT, VL3-BA, VSL2 and IUPRED software that consistently predicted the first 173 aa residues as an intrinsically disordered region (IDR) (Figure 2A,B). In addition, an LCR was predicted in the N-terminus using Motif Scan, which corresponds to a sequence rich in proline (P) residues from aa 23 to 101 (Figure 2A, inset). PTMs of DBP, together with the nucleic-acid binding capacity, the predicted IDR and the LCR may allow DBP to form multivalent interactions that promote LLPS as a potential driving force for formation of viral RCs. In support of these observations, a recently reported tool that predicts a protein’s potential for phase separation based on aa sequence, PSPredictor [64], the full length sequence of DBP, the predicted IDR in the N-terminus and the proline rich region (PRR) all showed a high score in their predicted ability to induce phase transitions (Figure 2C). In comparison, two cellular proteins that are known to drive phase separation, the FUS and DDX4 proteins, are assigned a score of 0.99 and 0.64, respectively, by PSPredictor [78], while the ACTB protein, a component of microfilaments that does not induce droplet formation is given a score of 0.0145. Therefore, the sequence analysis of DBP suggests that this viral early protein can be predicted to establish multivalent interactions and may be prone to induce condensation and assembly of RCs.

**Figure 3:**
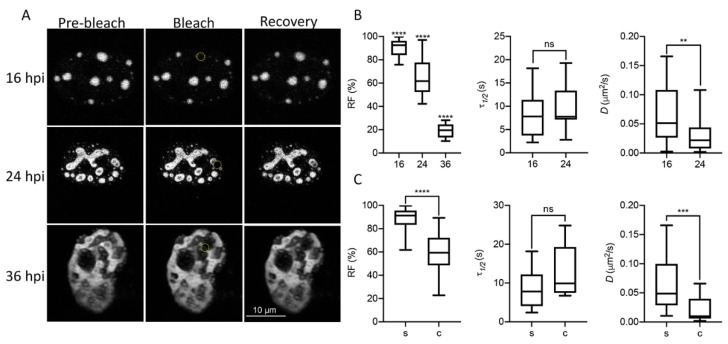
DBP displays biophysical features of proteins that drive phase transitions in infected cells. (**A**) Representative image frames of HFF cells infected with the mCherry-DBPv virus (MOI 30 FFU/cell) imaged at 16, 24 and 36 hpi for FRAP analyses. Pre-bleach, bleach and recovery frames, extracted from Supplementary Movies S7 (16 hpi), 8 (24 hpi) and 9 (36 hpi) are shown. The dotted yellow circles in the Bleach column show the bleached region. (**B**,**C**) Box-plots comparing the recovery fraction (RF), half-time of recovery (*τ*_1/2_) and diffusion coefficients (*D*) between time-points (**B**) or between spheroid (s) and complex (c) DBP-condensates (**C**). Statistical significance was calculated using a two-tailed Student’s *t*-test using GraphPad Prism 8. The significance of *p* values is shown as **** *p* < 0.0001; *** *p* < 0.001; ** *p* < 0.01. The boxes represent the distribution of the data and the whiskers go down to the smallest value and up to the largest. At least 16 cells per condition were analyzed. Details on the parameters and time-series can be found in supplementary data, Tables S1–S3 and Figures S1–S3.

In order to study liquid-like properties of DBP-assemblies, the mCherry-DBPv virus was used to infect HFF cells and live-cell imaging analyses were performed. First, we measured the diffusion coefficient of DBP by FRAP (Figure 3 and Supplementary Movies S7–S9) at three time-points post-infection: 16 hpi, which as described above corresponds to the early phase of viral replication in HAdV-infected HFF cells; 24 hpi, a time post-infection when an exponential increase in the accumulation of newly synthesized viral DNA molecules is observed; and 36 hpi, a late time-point when RCs have coalesced and viral progeny can be detected (see Figure 1A) (reviewed in [23]).

Figure 3A shows pre-bleach, bleach and recovery frames from representative cells at each time-point. At least 16 cells were evaluated for each time-point. The RF, which represents the mobile fraction that contributes to the recovery of fluorescence; the *τ_1/2_*, which is the time it takes for the curve to reach 50% fluorescence intensity between the bleached point and the plateau; and the diffusion coefficient, *D*, were measured to compare these parameters between time-points (Figure 3B), and between the spheroid (s) or complex (c) morphologies observed for DBP (Figure 3C) in infected cells. At 16 hpi, when DBP was mostly organized in spheroid foci (see Figure 1), the RF was almost 100%, and decreased at later times post-infection when more complex ring-like, fiber-like or amorphous structures were observed (Figure 3B,C). At 36 hpi, the average of the RF was almost null (Figure 3B) and therefore the *τ*_1/2_ and *D* could not be calculated. The *τ*_1/2_ increased to almost 20 s by 24 hpi for complex DBP-structures. However, although the average diffusion coefficient decreased by 24 hpi for non-spherical structures, the diffusion coefficients at this time-point are consistent with that of phase-separated organelles which range between 1 × 10^−5^ and 5 × 10^−1^ µm^2^/s [79,80]. The decrease in liquid-like properties of DBP-assemblies at later time-points suggests that they may undergo what has been called an ageing process, transitioning from liquid to gel or solid states, as has been reported for other liquid condensates [81,82].

**Figure 4:**
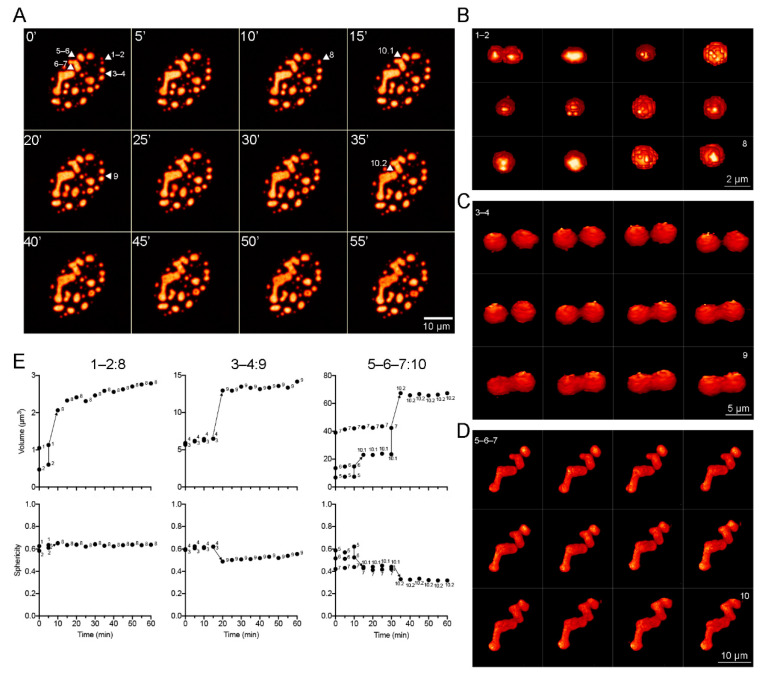
DBP condensates with distinct sizes and shapes fuse at different rates. HFF cells infected with the mCherry-DBPv virus (MOI 30 FFU/cell) were imaged from 20 to 24 hpi (Supplementary Movies s10 and s11) by spinning-disk confocal microscopy. (**A**) Montage of maximum intensity projections at 5 min intervals, extracted from Supplementary Movies S10 and S11. Arrowheads point to specific condensates (1–7) that fuse to form larger condensates (8–10) at different rates. (**B**–**D**) Montage of 3D reconstructions for condensates 1–2 (**B**), 3–4 (**C**) and 5–7 (**D**) illustrating the fusion process, extracted from Supplementary Movies S12, S13 and S14, respectively. (**E**) Dot-plots of the changes in volume and sphericity index as a function of time during the fusion processes. The color palette corresponds to the pixel gray level intensity under the Lookup Table NanoJ-Orange (Fiji), from dark orange to light orange.

To further determine if DBP-RCs display liquid-like properties within infected cells, time-lapse live-cell microscopy analyses were performed to study whether the RCs undergo fusion events, which are an additional characteristic of BMCs (Figure 4 and Supplementary Movies S10 and S11).

3D-reconstruction images were generated to qualitatively analyze the changes in morphology during fusion events. Figure 4A shows a montage of frames extracted from Supplementary Movies S10 and S11, where DBP condensates with different sizes and shapes that fuse together are indicated by arrowheads and numbers. Figure 4B–D show a montage of the 3D reconstruction of condensates indicated in Figure 4A. The volume and sphericity index of DBP condensates were measured directly from the Z-stacks (without rendering) and are presented as a function of time in the plots in Figure 4E. The spheroid condensates 1 and 2 (with volumes of approximately 1.1 µm^3^ and 0.5 µm^3^, respectively) fused within the first 10 min of imaging forming condensate 8 resulting in a similarly spheroid shape and a volume of around 2.5 µm^3^ (Figure 4B,E, left column: 1–2:8). Condensates 3 and 4 are similar in size, but larger than 1 and 2, and fuse within 20 min of imaging forming condensate 9 with a less spherical volume approximately 15 µm^3^ (Figure 4C,E, middle column: 3–4:9). Condensates 5 and 6 make contact and begin fusion during the first 15 min of imaging forming condensate 10.1, which then fuses with condensate 7 at 35 min resulting in an enlarged condensate of more than 60 µm^3^ (Figure 4D,E, right column: 5–6–7:10). Taken together, the data obtained by FRAP and time-lapse live-cell microscopy analyses from HAdV-infected cells show that DBP assembles liquid-like RCs that fuse and mature into larger and less fluid-like condensates.

### 3.3. Transient Expression of DBP Recapitulates the Assembly of RC-Like Liquid Condensates

**Figure 5:**
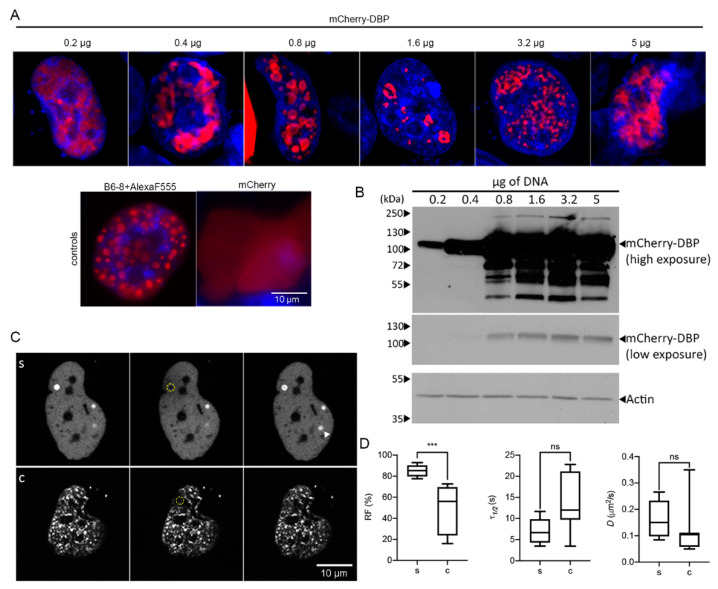
Transiently expressed DBP organizes in RC-like liquid condensates. H1299 cells were transfected with the specified amounts of mCherry-DBPp plasmid. (**A**) Confocal microscopy images showing the nuclear distribution of DBP. As controls, H1299 were transfected with a plasmid encoding the DBP (detected by indirect immunofluorescence using the primary antibody B6-8 and secondary anti mouse IgG coupled to Alexa Fluor 555) or with a plasmid encoding the mCherry protein. (**B**) Western blot from 50 µg of total protein lysates and staining of DBP. Actin was used as loading control. (**C**) Representative image frames of cells showing spheroid (s) or complex (c) DBP-condensates imaged for FRAP analyses. Pre-bleach, bleach and recovery frames, extracted from Supplementary Movies S15 (s) and S16 (c) are shown. The dotted yellow circles in the Bleach column show the bleached region. (**D**) The box-plots show the comparison between the RF, *τ*_1/2_ and *D* of s and c DBP-condensates. At least seven cells per condition were analyzed. Statistical significance was calculated using a two-tailed Student’s *t*-test using GraphPad Prism. The significance of *p* values is shown as *** *p* < 0.001. The boxes represent the distribution of the data and the whiskers go down to the smallest value and up to the largest. Details on the parameters and time-series can be found in supplementary data, Table S4 and Figure S4.

In addition to the live-cell studies of infected cells, we wished to determine if DBP was able to form droplet-like structures in a concentration-dependent manner in the absence of other viral molecules. Therefore, different amounts of the mCherry-DBPp plasmid, ranging from 0.2 µg to 5 µg, were transfected into readily transfectable H1299 cells (since primary HFF cells are not transfectable) and the cells were analyzed 24 hpt by single-cell confocal fluorescent microscopy (Figure 5).

The distribution of DBP was mainly diffuse in the nucleoplasm, although small spheroid droplets could be detected in the nucleus when 0.2 µg of plasmid DNA were used for transfection (Figure 5A). Surprisingly, with increasing amounts of plasmid more than 50% of the cells showed a droplet and ring-like distribution of DBP at sites that were depleted of DAPI staining. The distribution of DBP in these sites clearly resembles that of RCs at early and intermediate times post-infection in HAdV-infected cells. With the highest amount of DNA tested, DBP was still distributed in defined intranuclear structures. However, the morphology of the latter structures resembled that of RCs at late times post-infection with a coalesced or meshwork-like appearance. To determine whether the droplet and ring-like signals of DBP were not artifacts of the fusion with mCherry, we also evaluated the expression of mCherry alone, which showed a diffuse pattern in the entire cell (Figure 5A, controls). Moreover, transient transfection of the E2A gene and staining with the B6-8 antibody to detect DBP also revealed the droplet appearance of DBP inside the nucleus (Figure 5A, controls). Whole-cell lysates were used in immunoblot assays to analyze the mCherry-DBP protein steady-state levels expressed from different amounts of transfected plasmids (Figure 5B). The expected size of the fusion protein is approximately 100 kDa. DBP protein levels increased with increasing amounts of transfected DNA, especially between 0.2 to 3.2 µg. Lower and higher migrating bands were also detected with 0.8 µg or more DNA transfected (Figure 5B). Higher migrating bands could correspond to PTMs such as phosphorylation [56] or SUMOylation [55] while lower migrating bands could be proteolytic products of DBP [83]. These results indicate that in the absence of other viral molecules DBP can assemble RC-like structures in a concentration-dependent manner.

To measure the diffusion coefficient of DBP in RC-like condensates, FRAP analyses were conducted in transiently transfected cells (Figure 5C,D, Supplementary Movies S15 and S16). Data are presented comparing spheroid (s) and complex (c) morphologies as in Figure 3. As expected, the DBP that organized in spheroid structures had a higher recovery fraction. However, no significant difference was measured comparing the time of recovery and diffusion coefficients for s and c morphologies; nevertheless, the values obtained for these parameters were within those that have been reported for proteins that form BMCs (1 × 10^−5^–5 × 10^−1^ µm^2^/s) [79,80] (Figure 5D). A noteworthy observation was that in the short time frame of imaging (maximum 3 min) a DBP spheroid focus that was not bleached increased in intensity (Figure 5(Cs), arrowhead), in agreement with the rapid increase of DBP molecules within spheroid condensates.

Taken together, the data indicate that adenovirus DBP assembles BMCs with liquid-like properties and suggest that the formation of RCs could be driven by LLPS, a common mechanism for cellular compartmentalization and for the formation of an increasing number of virus-induced membrane-less organelles.

## 4. Discussion

In the present work, the liquid-like properties of DBP-positive RCs were evaluated. As described in the introduction, DBP is one of the main components of adenovirus RCs and participates not only directly in viral genome replication, but also in different aspects of viral mRNA biogenesis, activities that are also associated with RCs [23]. Some of the known features of DBP and the predictive bioinformatic tools used in this study provide evidence that DBP has features of proteins that induce phase separation: the protein binds both DNA and RNA [49,50,51,52]; contains an N-terminal region predicted to be intrinsically disordered and a proline-rich LCR, features that are characteristic of protein motifs and domains that are known to induce phase transitions (Figure 2) [84]. The FRAP and live cell microscopy analyses (Figure 1 and Figure 3) show that DBP-RCs are highly mobile, fuse between each other and mature over time to form less fluid-like arrays, when DBP becomes less mobile and ceases to interchange with the surrounding cellular milieu. Moreover, DBP has the ability to assemble RC-like structures in the absence of other viral proteins or nucleic acids as shown in the transient transfection assays (Figure 5). Collectively, these features represent the known hallmarks of BMCs that form by LLPS, and provide evidence that HAdV-RCs are “viral BMCs” that compartmentalize and concentrate viral and cellular macromolecules to promote efficient viral replication.

Alternative products of the E2A mRNA encode proteins with lower molecular weight than the 72 kDa major product produced during infection, and some of these products have been shown to bind ssDNA [53,75,83]. Phosphorylation at the N-terminal region of DBP modulates the ability of the protein to bind ssDNA [83], and SUMOylation of DBP is necessary for RC-assembly [55]. Moreover, two subsets of the 72 kDa DBP species have been defined by immunofluorescence and biophysical in situ fractionation [75]. One of these protein species displays diffuse nuclear staining, which closely resembles DBP at early times in the experiments shown in Figure 1 or when the protein is expressed at the lower concentrations of DNA (Figure 5); the second species displays a pattern of distribution that is similar to DBP-condensates observed at late times post-infection (Figure 1) or in cells transfected with higher amounts of DNA (Figure 5A,B).

With progression of the viral replication cycle, inner DBP-subcompartments are formed within the RCs, correlating the increase in the overall complexity of the morphology of DBP-condensates with a reduction in their fluid-like features (Figure 1, Figure 3 and Figure 4). Whether multiple condensates formed by LLPS coexist within RCs cannot be determined with these experiments and is currently under investigation. However, sites of active viral DNA replication and transcription are known to be spatially organized within RCs [18] and our experiments show that newly synthesized viral DNA is localized in the inner seemingly “hollow” spaces of DBP condensates (Figure 1B). Cellular BMCs can be organized in liquid-like subcompartments, as has been shown for the nucleolus [85], which allows to spatially separate distinct biochemical reactions. Moreover, it is proposed that RNA can modulate the organization and dynamics of BMCs, since increasing amounts of RNA can induce formation of subcompartmentalized condensates assembled by intrinsically disordered proteins [86,87]. Therefore, it will be interesting to determine if viral gene transcription or DNA replication, processes that will increase the concentration of nucleic acids within RCs, cause the subcompartmentalization of viral BMCs and if such subcompartments also possess liquid-like properties.

Chromatin and interchromatin compartments confine nuclear mobility of biomolecules and biomolecular complexes within the nucleus. Transiently transfected cells showed that DBP can condensate into RC-like structures, which could result from the protein’s ability to self-associate, as has been reported in in vitro analyses [88]. A surprising observation was that, similar to infected cells, these condensates were excluded from cellular chromatin, as the signal for DBP does not coincide with the DAPI staining (Figure 1 and Figure 5). HSV-1 infection has been shown to remodel the host chromatin structure to form RCs and to facilitate the diffusion of viral capsids during the assembly process [89]. Although the adenoviral assembly mechanism differs from that of herpesviruses, it is tempting to speculate that adenovirus infection may also remodel the host-chromatin architecture for efficient RC assembly and to facilitate the movement of molecules in and out of these sites. In agreement with this observation, it has been shown that some intrinsically disordered proteins mechanically exclude chromatin during assembly of BMCs [90] and that the chromatin mesh can define the size and distribution of these condensates [91].

Viral RCs can phase separate, fuse and coalesce, features that have been shown for membrane-less cellular compartments [92]. These properties are defined by the emergence of interfacial tension between different liquid phases. Nevertheless, what drives the auto-assembly or condensation of DBP and determines its interfacial tension with the surrounding nucleoplasm to form viral BMCs remains to be determined. Assembly of BMCs has been suggested to be driven by molecules that are referred to as “scaffolds”, which constitute the minimal component(s) necessary to induce condensation, while the rest of the components of BMCs are referred to as “clients” that are recruited through their interaction with the scaffolds [93]. LLPS must be promoted by a seeding or nucleation event that triggers the separation of two biochemically distinguishable phases by an increase in interactions and critical concentration of “scaffolds”. For cellular BMCs, seeding events for RNA granules have been shown to be induced by RNA, DNA or poly(ADP-ribose) [94,95,96]. Therefore, it will be interesting to identify the scaffolds and clients of HAdV-RCs to gain deeper insight into the molecular mechanism responsible for this viral compartmentalization strategy. The transient expression of DBP recapitulates the assembly and liquid-like properties of RC-like condensates, suggesting that DBP may be a scaffold protein (Figure 5). Various additional features of BMCs may shed additional details and should be explored further to unequivocally determine that DBP drives the formation of HAdV-RCs by LLPS; these may include for example the determination of the ability of purified DBP to form liquid condensates in vitro [97] and the effect of small aliphatic alcohols [98] and salt concentrations [99] to reversibly perturb or sustain the integrity of HAdV-RCs; however, the criteria to identify the requirements and the defining characteristics of LLPS are clearly evolving [40,100,101].

Work is in progress to study the seeding process of HAdV-RCs and the scaffold and client components that are recruited there. The study of additional distinct biophysical features of DBP in the nucleoplasm versus the RCs will be necessary to define the role of DBP on RC-formation. In addition, evaluating other potential seeding or scaffold components of HAdV-RCs, such as nucleic acids or other proteins, in the formation of condensates will also contribute to understand whether LLPS may be the driving force in their assembly. This is especially important since, depending on the molecule analyzed, a BMC could display various liquid-like properties [100,101,102]. For example, HSV-1 RCs are membrane-less intranuclear compartments that share features with phase separated BMCs [103]. When studying ICP4, this viral protein displayed liquid-like properties that supported a LLPS-mediated mechanism for the formation of RCs [42]. However, recruitment of the RNA polymerase II to HSV-1 RCs appears to depend on a mechanism that does not implicate LLPS [102]. This evidence raises the possibility that viral RCs as well as other cellular compartments may be organized in subcompartments with different biophysical features.

Since several intracellular pathogens, including viruses, are known to induce the formation of intracellular liquid-like compartments and since failure to control intracellular condensation may lead to diseases, including cancer [104,105], continued studies of virus-induced cellular compartmentalization should shed light on fundamental aspects of virus-host interactions.

## Supplementary Materials

The following supplementary movies are available online at https://www.mdpi.com/article/10.3390/v13091778/s1, Figure S1. Plot of the time series for all cells analyzed by FRAP, 16 hpi (the frame rate is shown in the table). Figure S2. Plot of the time series for all cells analyzed by FRAP, 24 hpi (the frame rate is shown in the table). Figure S3. Plot of the time series for all cells analyzed by FRAP, 36 hpi (the frame rate is shown in the table). Figure S4. Plot of the time series for all transfected cells analyzed by FRAP (the frame rate is shown in the tables). Table S1. FRAP parameters for infected cells, 16 hpi. Table S2. FRAP parameters for infected cells, 24 hpi. Table S3. FRAP parameters for infected cells, 36 hpi. Table S4. FRAP parameters for transfected cells. Movies 1–16. FRAP parameters and time-series plots can be found in Supplementary data.
