# Supplementary material for: Evidence That the Adenovirus Single-Stranded DNA Binding Protein Mediates the Assembly of Biomolecular Condensates to Form Viral Replication Compartments
Source: Viruses. 2021 Sep 6;13(9):1778. doi: 10.3390/v13091778 (PMC8473285; doi:10.3390/v13091778)
Supplement: Supplementary file 1 [file viruses-13-01778-s001.zip › Supplementary data.pdf]

Article

The Adenovirus Single-Stranded DNA Binding Protein Mediates the Assembly of Biomolecular Condensates through Liquid-Liquid Phase Separation to Form Viral Replication Compartments.

Paloma Hidalgo[1,2,#,*], Arturo Pimentel[3,#], Diana Mojica-Santamaría[1], Konstantin von Stromberg[2], Helga Hofmann-Sieber[2], Christian Lona-Arrona[1,4], Thomas Dobner[2] and Ramón A. González[1,*]

Supplementary Data

FRAP parameters for infected (16, 24 and 36 hpi) and transfected cells and time series plots.

RF: recovery fraction
τ ½: half-time of recovery
D: diffusion coefficient
ROI: Region of interest
fps: frames per second
s: spheroid
c: complex

Table S1. FRAP parameters for infected cells, 16 hpi

cell	RF (%)	τ 1/2 (s)	D ($\mu m^2/s$)	Bleaching (%)	Bleach ROI size (µm)	Morphology	Frame rate (fps)	Frames pre-bleach	Bleach Frame	Bleach duration (ms)	Total frames
1	94.20	2.38	0.166	72.4	0.965	s	0.45	29	30	200	350
2	83.70	3.64	0.121	80.2	0.965	s	0.45	29	30	200	350
3	83.20	3.99	0.1107	78.4	1.104	s	0.45	29	30	200	350
4	95.60	11.87	0.0372	78.2	1.104	s	0.45	29	30	200	350
5	81.00	150.56	0.0026	68.1	1.104	c	0.45	29	30	200	350
6	79.90	9.91	0.039	70.5	1.104	c	0.45	29	30	200	350
7	98.50	3.49	0.113	72.8	0.965	s	0.45	29	30	200	350
8	85.20	7.83	0.0564	76.3	1.104	s	0.45	29	30	200	350
9	92.60	4.76	0.101	85.7	1.104	s	0.45	29	30	200	350
10	91.50	13.42	0.0329	76.2	1.104	s	0.45	29	30	200	350
11	97.30	8.51	0.0519	78.3	1.104	s	0.45	29	30	200	350
12	93.20	18.16	0.0243	75.3	1.104	s	0.45	29	30	200	350
13	92.50	16.39	0.0241	68.9	1.104	s	0.45	29	30	200	350
14	95.40	9.59	0.0505	90.2	1.104	s	0.45	29	30	200	350
15	75.80	45.57	0.0106	90.6	0.965	s	0.45	29	30	200	350
16	90.30	4.09	0.096	68.5	0.965	s	0.45	29	30	200	350
17	99.70	13.2	0.036	83.7	1.104	s	0.45	29	30	200	350

18	96.5<br>0	7.77	0.062	91.6	1.104	s	0.45	29	30	200	350
19	88.9<br>0	31.66	0.0153	84.1	1.104	s	0.45	29	30	200	350
20	98.0<br>0	2.23	0.148	36.3	1.104	c	0.45	29	30	200	350

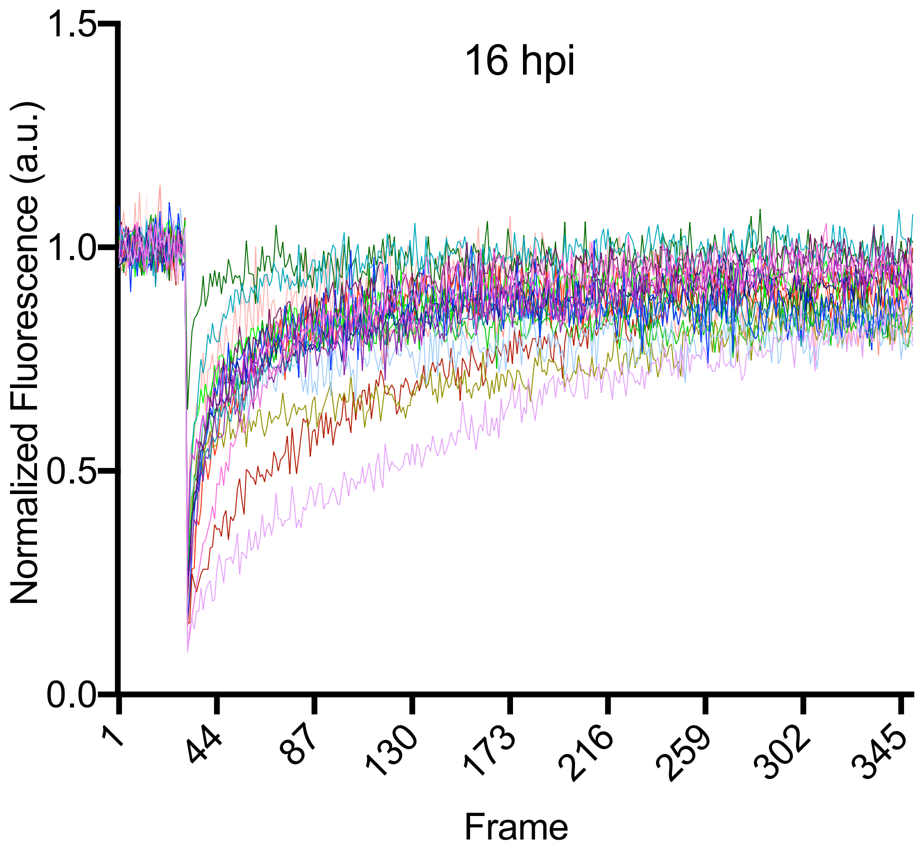

Fig S1. Plot of the time series for all cells analyzed by FRAP, 16 hpi (the frame rate is shown in the table).

Table S2. FRAP parameters for infected cells, 24 hpi

ce ll	RF (%)	τ 1/2 (s)	D (µm²/s)	Bleaching (%)	Bleach ROI size (µm)	Morpholo gy	Frame rate (fps)	Frames pre-bleach	Bleach frame	Bleach duration (ms)	Total frames
1	0.97	2.8	0.10808	69.8	0.965	s	0.56	19	20	100	120
2	0.91	9.47	0.031	73.8	0.965	s	0.2159	19	20	100	300
3	0.81	14.81	0.02	73.9	0.965	s	0.2159	19	20	100	300
4	0.74	8.12	0.0415	77.6	0.965	c	0.1889	19	20	100	300
5	0.66	7.47	0.04	72.9	0.965	c	0.1889	19	20	100	300
6	0.59	13.36	0.022	68.7	0.965	c	0.1889	19	20	100	300
7	0.50	19.28	0.017	77.9	0.965	c	0.1889	19	20	100	300
8	0.69	7.77	0.047	85.3	0.965	s	0.1889	19	20	100	300
9	0.74	7.13	0.042	74.2	0.965	s	0.1889	19	20	100	300
10	0.68	7.45	0.04	72.3	0.965	c	0.1889	19	20	100	300
11	0.62	11.7	0.028	75.2	0.965	s	0.1889	19	20	100	300
12	0.52	18.14	0.066	72.4	0.965	c	0.3268	19	20	100	300
13	0.89	7.3	0.046	79.3	0.965	c	0.1886	19	20	100	300
14	0.84	6.74	0.054	89.5	0.965	c	0.1886	19	20	200	300
15	0.89	4.54	0.074	82	0.965	s	0.56	19	20	400	250
16	0.61	36.3	0.01	92.7	0.965	c	0.1886	19	20	400	250
17	0.49	40.16	0.0092	93.2	0.965	c	0.1886	19	20	400	250
18	0.53	177.41	0.002	93.7	0.965	c	0.1886	19	20	400	250
19	0.53	55.12	0.0067	96.9	0.965	c	0.1886	19	20	400	300
20	0.72	21.88	0.0169	95.1	0.965	c	0.1886	19	20	400	300
21	0.47	94.68	0.0039	87.2	0.965	c	0.1886	9	10	100	250
22	0.57	39.21	0.0094	94	0.965	c	0.1886	19	20	400	300
23	0.42	77.63	0.004	90.4	0.965	c	0.1886	19	20	400	300
24	0.43	77.3	0.004	95.4	0.965	c	0.1886	19	20	400	300

25	0.53	55.12	0.0067	96.9	0.965	c	0.1886	19	20	400	300

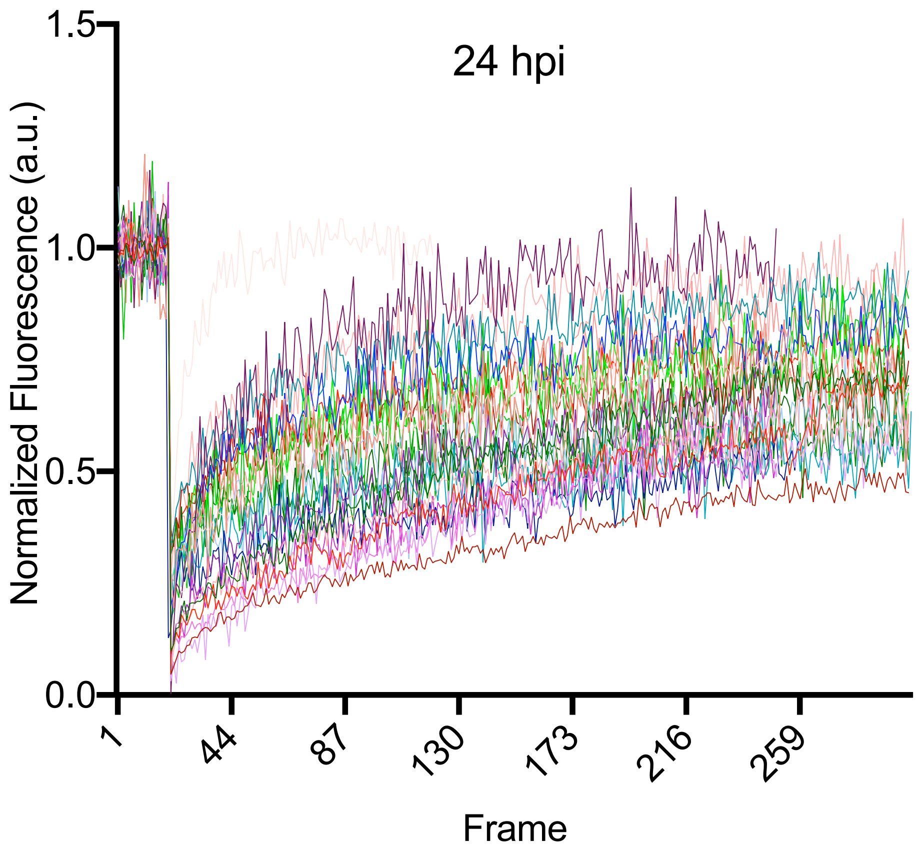

Fig S2. Plot of the time series for all cells analyzed by FRAP, 24 hpi (the frame rate is shown in the table).

Table S3. FRAP parameters for infected cells, 36 hpi

cell	RF (%)	τ 1/2 (s)	D (μm²/s)	Bleaching (%)	Bleach ROI size (μm)	Morphology	Frame rate (fps)	Bleach frame	Frames pre-bleach	Bleach duration (ms)	Total Frames
1	13.40	75.4		75.4	1.104	c	0.568	30	29	200	300
2	24.50	73.7		73.7	1.104	c	0.568	30	29	200	300
3	13.40	91.5		91.5	1.104	c	0.568	30	29	200	300
4	15.40	86.1		86.1	1.104	c	0.568	30	29	200	350
5	20.10	79.6		79.6	1.104	c	0.568	30	29	200	350
6	28.10	89.2		89.2	1.104	c	0.568	30	29	200	350
7	26.90	87.6		87.6	1.104	c	0.568	30	29	200	350
8	15.50	90.9		90.9	1.104	c	0.568	30	29	200	350
9	21.00	83.6		83.6	1.104	c	0.568	30	29	200	350
10	10.30	91.5		91.5	1.104	c	0.568	30	29	200	350
11	12.20	89.1		89.1	1.104	c	0.568	30	29	200	350
12	27.60	82.7		82.7	1.104	c	0.568	30	29	200	350
13	19.50	87.9		87.9	1.104	c	0.568	30	29	200	350
14	17.30	85.5		85.5	1.104	c	0.568	30	29	200	350
15	35.90	24.8	0.0195	85.1	1.104	c	0.568	30	29	200	350
16	22.80	62.5	0.0077	98.9	1.104	c	0.568	30	29	200	350

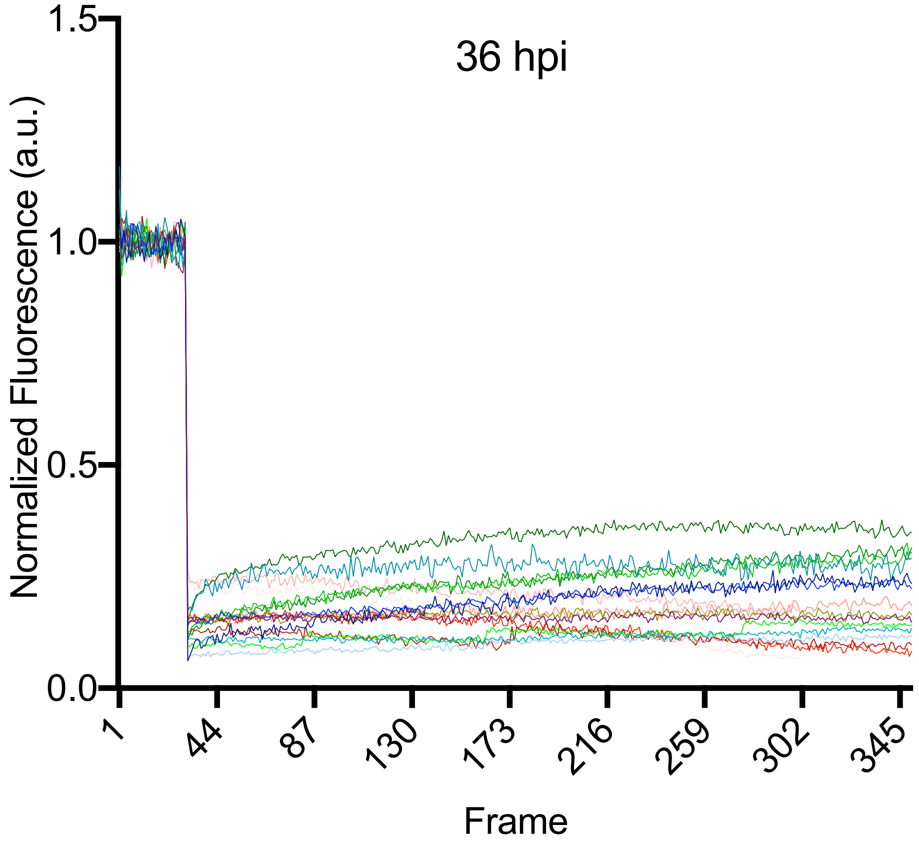

Fig S3. Plot of the time series for all cells analyzed by FRAP, 36 hpi (the frame rate is shown in the table).

Table S4. FRAP parameters for transfected cells

Spheroid distribution (s)										
Cel l	Bleach ROI (μm)	% Bleach	RF	τ 1/2 (s)	D (um²/s)	Frame rate (fps)	Frames pre-bleach	Bleach Frame	Bleach duration (s)	Total frames
1	1.828	60.4	83. 3	4.83	0.211011	1.0009	4	5	1	66
2	1.828	55.1	82. 5	11.68	0.08439	1.0009	4	5	1	66
3	1.828	43	92. 9	8.6	0.10976	1.0009	4	5	1	66
4	1.828	44.3	87. 2	8.34	0.11318	1.0009	4	5	1	66
5	1.828	51.4	88	9.8	0.09803	1.0009	4	5	1	66
6	1.828	48.4	92. 3	9.94	0.09665	1.0009	4	5	1	66
7	1.828	59	77. 5	4.4	0.23163	1.0009	4	5	1	66
8	1.828	38.3	89. 6	4.96	0.18695	1.0009	4	5	1	66
9	1.828	41.4	80. 3	3.49	0.26569	1.0009	4	5	1	66
10	1.828	42.8	77. 9	3.98	0.23718	1.0009	4	5	1	66
More complex distribution (c)										
cell	Bleach ROI (μm)	% Bleach	RF	τ 1/2 (s)	D (um²/s)	Frame rate (fps)	Frames pre-bleach	Bleach Frame	Bleach duration (ms)	Total Frames
1	1.828	62.6	69. 6	21.16	0.04974	1.0009	4	5	1	66
2	1.828	64	53	16.03	0.06566	1.0009	3	4	1	116
3	1.828	68.9	58. 4	9.72	0.11172	1.0009	3	4	1	95
4	1.828	86.1	23. 7	22.8	0.05825	1.0009	3	4	1	95

5	1.828	92.1	16	12.02	0.1105	1.0009	3	4	1	65
6	1.828	76.6	56	3.46	0.35	1.0009	3	4	1	95
7	1.828	76.3	72.<br>6	11.63	0.104155	1.0009	3	4	1	95

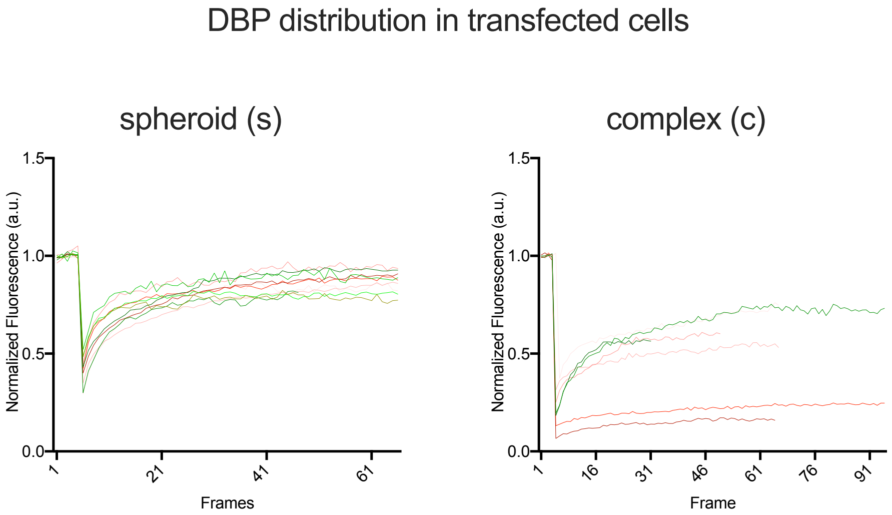

Fig S4. Plot of the time series for all transfected cells analyzed by FRAP (the frame rate is shown in the tables).
