# Supplementary figures and images for: Evidence That the Adenovirus Single-Stranded DNA Binding Protein Mediates the Assembly of Biomolecular Condensates to Form Viral Replication Compartments
Source: Viruses. 2021 Sep 6;13(9):1778. doi: 10.3390/v13091778 (PMC8473285; doi:10.3390/v13091778)

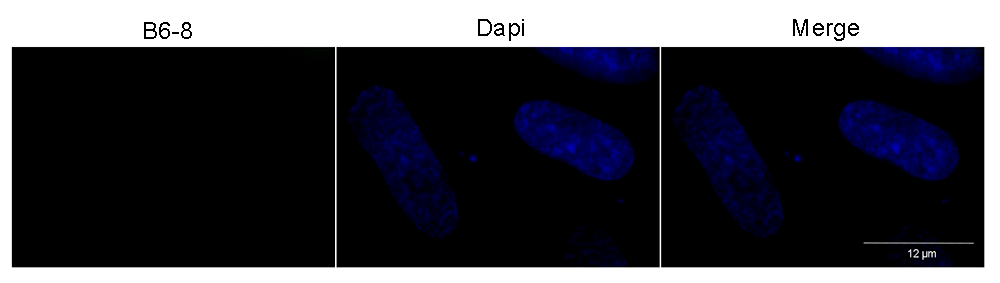

Supplement: Supplementary file 1 [file viruses-13-01778-s001.zip › Montage HFF Mock-infected B6-8 DAPI.tif]
